# Vitrification of Lepidopteran Embryos—A Simple Protocol to Cryopreserve the Embryos of the Sunflower Moth, *Homoeosoma electellum*

**DOI:** 10.3390/insects13100959

**Published:** 2022-10-20

**Authors:** Arun Rajamohan, Jarrad R. Prasifka, Joseph P. Rinehart

**Affiliations:** 1 Insect Biochemistry and Genetics Research Unit, USDA-ARS-Edward T. Schafer Agricultural Research Center, 1616 Albrecht Blvd., Fargo, ND 58102, USA; 2 Sunflower and Plant Biology Research Unit, USDA-ARS-Edward T. Schafer Agricultural Research Center, 1616 Albrecht Blvd., Fargo, ND 58102, USA

**Keywords:** lepidoptera, cryopreservation, sunflower moth

## Abstract

**Simple Summary:**

Since originally being described for fruit flies in 1990, insect embryo cryopreservation protocols have been developed for several fly species and have been used to preserve several important fly species and strains. However, the development of similar protocols for other types of insects has not progressed as rapidly. For instance, only three species of moths have been successfully cryopreserved, and the technique has not been widely adopted for any of these species. This is mainly due to complications associated with the outer egg coatings surrounding the developing moth embryo, which are substantially different from what surrounds a fly embryo. To expand the usefulness of this technique for moths, we have developed a cryopreservation protocol for the sunflower moth, a key pest of cultivated sunflower in the southern Great Plains of the United States. Our final protocol resulted in 23% of embryos hatching after cryopreservation, with 60% surviving to the adult stage. While designing this protocol, we also developed a novel method to quantitatively assess the ability of water to leave the egg after treatments, which is critically important to successful cryopreservation.

**Abstract:**

Embryos of the sunflower moth, *Homoeosoma electellum* (Hulst), were cryopreserved after modification to the method that was previously described for *Pectinophora gossipiella*. The workflow to develop the protocol consisted of methods to weaken the embryonic chorion followed by the application of various methods to disrupt the sub-chorionic wax layer. These steps were necessary to render the embryos permeable to water and cryoprotectants. Initially, the embryos were incubated at 21° and 24 °C, and the development of the double pigment spots/eyespot and eclosion were tracked every two hours. The embryos at 24 °C showed eyespots as early as 30 h, while in the case of the embryos that were incubated at 21 °C, there was a developmental delay of approximately 20 h. The embryos at 24 °C showed peak eclosion between 55 and 70 h, and the embryos at 21 °C eclosed between 80 and 100 h of development. Estimating this range is crucial for the purposes of stage selection and treatment initiation for cryopreservation protocol development for the embryos. The control hatch percentage at either developmental temperature was >90%, and the sodium hypochloride, 2-propanol and alkane-based treatments reduced the embryo hatchability to <10%. Hence, a modified surfactant—hypochlorite mixture—was used to destabilize the chorion and solubilize the hydrophobic lipid layers. Water permeability assessments using the dye-uptake method show that polysorbate 80 in combination with sodium hypochlorite alone is capable of permeabilizing the embryo as efficiently as sequential hypochlorite—alkane treatments, but with significantly higher hatch rates. A vitrification medium consisting of ethane diol and trehalose was used to dehydrate and load the embryos with the cryoprotective agent. The median hatch rates after vitrification were 10%, and maximum was 23%.

## 1. Introduction

Germplasm conservation of both economically and ecologically important insect species is crucial for protecting the biosphere, as well as human interests. Numerous past studies have observed that the germplasm conservation of ‘deemed’ inimical species such as the pests of crops and vectors of diseases is as important as the conservation of endangered species [1]. These species are the subject of extensive research to better comprehend their biology and to develop effective control techniques which would reduce the ecological impacts of the current chemical control practices. These studies require a constant supply of the species of interest, which is both costly and carries risks of failure. The ability to extract live organisms from cryostorage to resurrect both small- as well as large-scale colonies reduces many of the risks associated with the continuous mass culture of organisms [2].

The sunflower moth, *Homoeosoma electellum* (Hulst) (Lepidoptera: Pyralidae), is a key pest of cultivated sunflowers in the southern Great Plains of the United States. After adults oviposit on blooming sunflowers, larvae hatch and feed sequentially on pollen, florets and developing seeds. Each larva developing on a sunflower can be extremely destructive, feeding on more than 90 florets and 20 seeds [3]. Poor cold tolerance in the larval stages of this species suggests that successful overwintering cannot occur north of Kansas [4], despite the evidence of the occurrence of cryoprotectants in the diapausing larval stage(s) [5].

Cryobiological germplasm conservation studies on lepidopterans are meager, despite the economic importance of members of this order [6,7,8,9]. Previously, we described the durability of lepidopteran eggs (embryos), especially their ability to withstand both natural and artificial attempts to dehydrate them at any developmental stage prior to larval eclosion [8]. This characteristic is primarily responsible for stymieing cryopreservation conservation studies on lepidopteran embryos/eggs, as chorion forms the primary environmental and predatory barrier in many families of insects [10,11]. On the other hand, numerous cryopreservation studies have been conducted on dipterans which take advantage of the functional but flimsy chorion, leaving the primary cryopreservation challenge of the wax barrier overlying the vitelline membrane, as noted by Steponkus [12].

In this report, a cryopreservation protocol for *Homoeosoma electellum* is presented and compared with a protocol that was previously reported for the economically important pink bollworm, *Pectinophora gossypiella* [8]. This study is an assessment of the parameters that make the sunflower moth embryos resistant to permeabilization and post-permeabilization treatments. Initially, the embryos that were collected from caged moths were allowed to develop at two different temperatures to determine the specific developmental stages that are amenable to cryopreservation. Thereafter, the embryos were assessed for tolerances to commonly used dechorionating agents and surface-delipidating solvents, the effectivenesses of which were determined by embryonic viability and changes in their ability to uptake water and marker dye. Embryos that were deemed to be permeabilized were assayed for cryoprotective agent (CPA) tolerance and uptake, as well as dehydration susceptibility. Finally, viable embryos after each treatment were allowed to hatch, pupate and eclose as adults, with the proportion of resultant viable life stages used to define the successfulness of the designed protocol.

## 2. Materials and Methods

### 2.1. Insect Source, Rearing Method and Egg Collection

Sunflower moth (*Homoeosoma electellum* (Hulst)—Pyralidae) colonies were established using wild-collected larvae. During summer of 2013, wild and cultivated sunflower heads with visible frass and webbing were collected from central and western North Dakota. In the lab, sunflower moth larvae were manually removed and placed into individual cups (to minimize the transmission of any pathogens) containing a wheat-germ-based artificial diet. The diet was adapted from Wilson [13], with a small amount of aureomycin (0.25 g/100 g of dry ingredients) added. Cups with larvae were placed in an environmental chamber set to 28 °C, 70% RH, with a 14:10 L:D cycle. After pupae were visible in the cups, the pupae were moved into a mesh cage in the same environmental chamber. After the first adults emerged, they were provided cotton wicks moistened with 10% sucrose. Starting 5 days after emergence began, the moths were provided with wax paper strips folded into a ‘V’ shape nested in foam blocks and dusted with pollen, a method that provides both chemical and physical cues needed to induce oviposition [14]. The environmental chamber settings (28 °C, 70% RH, 14:10 L:D) produced a generation time (egg-to-egg) of 30 days and prevented entry into diapause. After two generations, larvae were reared collectively in large dishes rather than individual cups and crawled to pupate inside wax-coated cardboard rings, as described by Wilson [13].

### 2.2. Egg Collection Conditions for Treatments

Moths, which were maintained in an environmental chamber at 28 °C, were permitted to lay eggs in a folded ‘V’-shaped wax paper dusted with sunflower pollen as described above for a period of 1–2 h. After the egg collection, the embryonic developmental synchronicity and hatchability were assessed at two incubation temperatures, 21.0 and 24.0°C, in the dark, with unmanaged relative humidity (RH) ranging from 20–55% (Echotherm in30 incubator, Torrey Pines, Carlsbad, CA, USA). The developmental status of the embryos was assessed after 48 h, after which the embryos were further processed for permeabilization and freezing. The number of embryos that had developed the dual pigmented spots, termed as ‘*eyespot*’ in this study, as well as the number of embryos that had eclosed, were noted after oviposition every 120 min for 6 h after 24, 48, 72, 96 and 120 h.

### 2.3. Permeabilization

Our embryonic permeabilization procedure initially followed the routine procedure described in Wang et al. [15] for dipterans, and thereafter a modified procedure described in Rajamohan et al. [8] for lepidopterans. Both procedures are multi-step process involving the destabilization of the chorion followed by dissolution of the natural wax layer between the inner chorionic layer and the vitelline membrane. However, prior to permeabilization the eggs were dislodged from the wax paper by soaking the pieces of the paper containing the eggs in 1% sodium hydroxide solution for one minute, followed by rinsing with the same solution until the eggs were dissociated from one another [16]. Eggs were then immediately filtered from the medium using a 150 µm nylon mesh basket and rinsed for one minute in running tap water. The developmental stage of the embryo was assessed as described in Rajamohan et al. [8]. Each permeabilization method was then assayed as described below.

As in the method based on dipteran studies, the wet embryos were suspended in a 20–25% aqueous solution of sodium hypochlorite (Sigma-Aldrich, St. Louis, MO, USA) and continuously agitated over a period of 120 s. Embryos withdrawn 90 and 120 s were thoroughly rinsed and assessed for the chorionic stability. This was followed by 15–20 s of surface dehydration, hereafter referred to as de-wetting, using 2-propanol (99.9% purity; Sigma-Aldrich, St. Louis, MO, USA). Following one minute of air drying, the embryos were rinsed in two nonpolar solvents, namely hexanes (Burdick & Jackson, Muskegon, MI, USA) and heptane (Sigma-Aldrich, St. Louis, MO, USA), to remove the wax. They were then briefly air dried to remove residual solvents and resuspended in an aqueous near-isotonic mediums of silkworm Ringer [860 mg NaCl, 10 mg KCl and 33.3 mg CaCl2 in 4:6 sodium (dibasic): potassium (monobasic) phosphate buffer, pH 6.6] or Grace’s cell culture medium (Gibco/Thermo-Fisher, Waltham, MA, USA).

In the methods based on lepidopteran studies, a surfactant, 0.5% polysorbate 80 [P80; polyoxyethylene sorbitan monooleate] (Sigma-Aldrich, St. Louis, MO, USA), was used both with and without 10% sodium hypochlorite to permeabilize the embryos that were first blotted to remove excess water after the dissociation treatment in sodium hydroxide. The embryos were treated for between 120 to 300 s and assessed for viability and permeability as described below.

### 2.4. Permeability

Embryonic permeability was assessed using 10µM aqueous rhodamine B (Mallinckrodt, St. Louis, MO, USA) [17]. A 100 µM stock solution was prepared in 5% ethanol [18]. The control and treated embryos were stained for one minute, rinsed in saline for one more minute, surface air dried for another minute and then placed on a coverslip, submerged in low-autofluorescence immersion oil (Olympus, Center Valley, PA, USA) and sealed in a cavity slide. They were then imaged at 469/525 nm (Ex/Em) on a Lionheart Automated Live Cell Imager (Agilent-Biotek, Santa Clara, CA, USA), and the stain/fluorescence intensity of each embryo was measured using the pixel intensity tools in Gen5 image capture and analysis software (Agilent, Santa Clara, CA, USA). Unstained 55–60 h old embryos and 0.1 µL droplets of 0.1% rhodamine B under the immersion oil served as a negative and positive control, respectively, and the observed intensities were used to set the intensity scale between 0.0 to 1.0.

### 2.5. Vitrification and Recovery

Our vitrification protocol was a modified method originally described by Rajamohan et al. [8]. Embryos that were between 45–55 h of development at 21 °C (which we determined to be the optimal stage for cryopreservation) were permeabilized using P80 containing 10% sodium hypochlorite for up to 180 s (which was determined to be the optimal permeabilization protocol). The permeabilized embryos were incubated for 12 or 15 min in a mixture of 6.45 M 1,2-ethanediol prepared with a solution of 0.5 M trehalose in Grace’s cell culture medium. A 5–10 µL drop containing ~70–150 embryos was placed as a uniform layer on top of a Whatman Nuclepore™ polycarbonate membrane (25 mm diameter; 8 µm pore; PVA coated; Millipore-Sigma, Burlington, MA, USA). After quickly blotting excess vitrification media through the membrane using low-lint wipes, the embryos were swiftly exposed to liquid nitrogen vapor for one minute, after which the membranes containing the vitrified embryos were placed in a histological cassette (Electron Microscopy Science, Hatfield, PA, USA) which was then stored submerged in liquid nitrogen in storage Dewars (MVE, Montevideo, MN, USA).

To recover the embryos, membranes were retrieved from the histology cassette while it was submerged in liquid nitrogen and then held in liquid nitrogen vapor for one minute. Thence the membrane was immediately plunged, embryo side down, into 3–5 mL of 0.5 M trehalose in Grace’s cell culture medium in a 3 cm sterile Falcon^®^ petri plate (Corning, Corning, NY, USA). The strainer containing the embryos was placed on a lint-free absorbent tissue paper to remove excessive thawing medium around the embryos. These embryos also tended to float when the strainer was placed gently in the cell culture medium. Embryos that were encouraged to float tended to prevent submerged eclosion and drowning, despite the buoyant nature of the larvae.

### 2.6. Post Treatment Rearing

Larvae that hatched after each treatment, including vitrification, were usually found floating on the cell culture medium. These larvae were transferred to a larval-rearing medium in plastic cups and reared to pupation and eclosion as previously described [13].

### 2.7. Data Analysis

All of the data generated in this study are expressed as proportions and assessed as binomial distributions. Therefore, the data was modeled using generalized linear modeling or compared using non-parametric comparative assessments, such as the Kruskal–Wallis equality-of-populations rank test. Multiple comparisons were performed using Dunn’s test with the Benjamini–Yekutieli adjustment available in the statistical software Stata IC v16.1 (College Station, TX, USA) [19,20].

## 3. Results

### 3.1. Developmental Staging by Temperature

**Figure 1:**
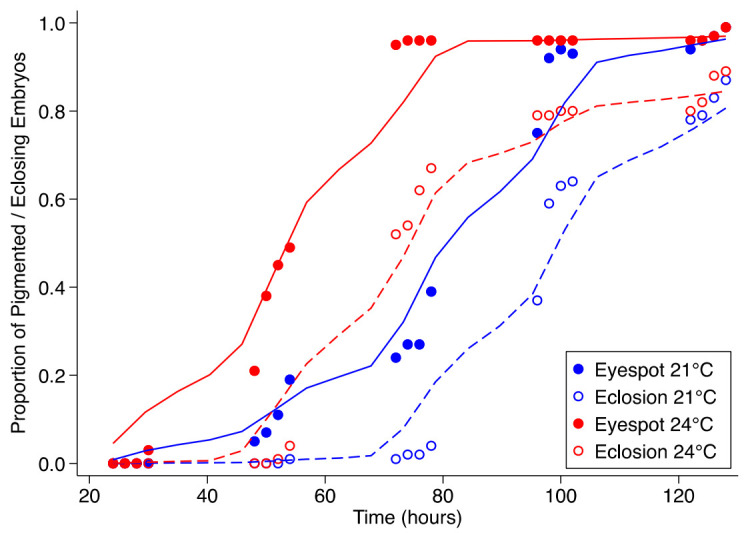
Proportion of sunflower moth embryos with pigmented eyespots (closed circles) and the proportion that eclosed (open circles) during incubation at 21 °C (blue) and 24 °C (red).

As expected, embryos assessed after 48 h at either 21 °C or 24 °C were distinctly different in appearance. However, there were no significant differences in the synchronicity of eyespot development or eclosion (Figure 1). Embryos were distinctly segmented after 30 h at 24 °C and ~50 h when incubated at 21 °C. Pigmented eyespots were observed at about 5–10 h after the segmentations were observed. The pigmented spots appeared in the embryos at a mean time point of 35.5 ± 9.2 h and 46.3 ± 4.8 h in embryos developing at 24° and 21 °C, respectively. At 21 °C, larval emergence was noticed after 75–80 h, while emergence was noticed after 55 h at 24 °C. Hence, the embryos that were incubated in the lab at 21.0 and 24.0 °C showed initial eclosion nearly 24 h apart, and the treatment times were also similarly affected.

### 3.2. Permeabilization

**Figure 2:**
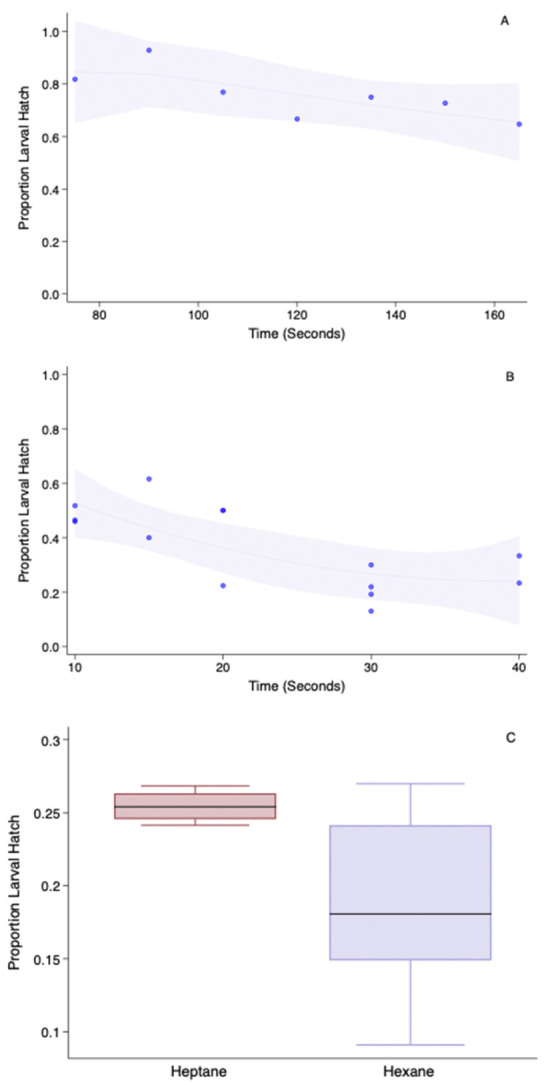
Effect of permeabilization steps on sunflower moth larval eclosion rates, including (**A**) dechorionation treatment using 25% sodium hypochlorite for up to 3 min, (**B**) surface dehydration by treatment with 2-propanol for up to 40 s after 160 s in sodium hypochlorite solution and (**C**) removal of the wax layer with either heptane or hexanes for 40 s after 160 s in sodium hypochlorite and 20 s in propanol. In (**A**,**B**), the line fits the predicted value from a quadratic regression model superimposed over the scatter plot of the data. The grey shaded area represents a 95% confidence interval from the quadratic model.

Permeabilization is a multistep process and involves the dechorionation and solubilization of hydrophobic wax layers. Figure 2A,B shows the non-adjusted viability of the embryos when treated with sodium hypochlorite (2A) for between 80 to 160 s and then sequentially followed by 100% 2-propanol (2B) for 10–40 s. Figure 2C depicts the adjusted embryonic hatch post heptane or hexanes treatment for 40 s. The approximate proportion of lost embryos due to the chlorine toxicity was about 0.35 (not adjusted for control larval eclosion) after 160 s. The hatch proportion was further reduced by 2-Propanol to 0.4 ± 1.6 after 20 s. The most commonly used wax/lipid clearing agents are alkanes such as hexanes or heptane. When the embryos were treated with 2-propanol for 20 s and dried for a minute and then treated with either of the alkanes for 40 s, the heptane-treated embryos suffered less damage, as noted by its higher hatch rate than the hexanes-treated embryos. However, they were not statistically different (H = 2.557, *p* = 0.11,) although the proportion median reduction was >0.7.

### 3.3. Permeability

**Figure 3:**
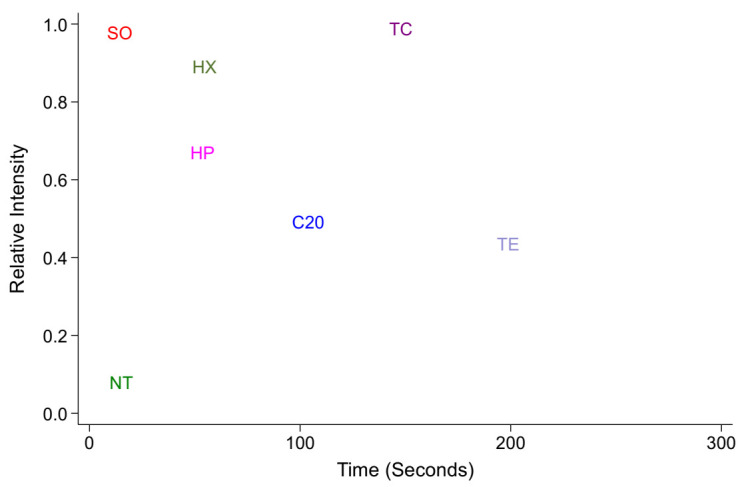
Effect of permeabilization treatments on the relative fluorescence intensities of the embryos stained with 10 µM rhodamine B dye. SO—0.2 µL of 0.1% rhodamine B aqueous solution; HX—hexanes-treated embryos; HP—heptane-treated embryos; C20 embryos were rinsed in 20–25% sodium hypochlorite; TE embryos were rinsed in 0.5% polysorbate 80; TC shows the fluorescent intensities of the embryos treated with 0.5% polysorbate 80 in 10% sodium hypochlorite.

The fluorescence intensity of rhodamine B that had penetrated the embryonic vitelline membrane and permeated through the embryo after permeabilization treatments was measured against positive and negative calibration intensities, which were repeated prior to each treatment (Figure 3). The rhodamine permeation rates were estimated for embryos treated with 20% sodium hypochlorite (marked C20 in Figure 3), 0.5% polysorbate 80 (marked TE in Figure 3) and 0.5% polysorbate 80 with 10% sodium hypochlorite (marked TC). The permeation intensity of the embryos treated with TC approached the intensity of positive control droplets with increasing time (120 s: H = 3.85, *p* = 0.049; 180 s: H = 0.8, *p* = 0.33). Timed assessments were not conducted for hexanes and heptane. Hexanes treatment for 40 s resulted in a slightly lower intensity than the TC treatment for 120 s (0.82 ± 0.05 versus 0.92 ± 0.03; H = 4.27, *p* = 0.039). Hexanes-treated embryos also exhibited higher uptake of the dye compared with the heptane-treated embryos, but they were not statistically different (H = 2.82, *p* = 0.093).

### 3.4. Cryoprotectant Tolerance, Post-Vitrification Viability and Eclosion

**Figure 4:**
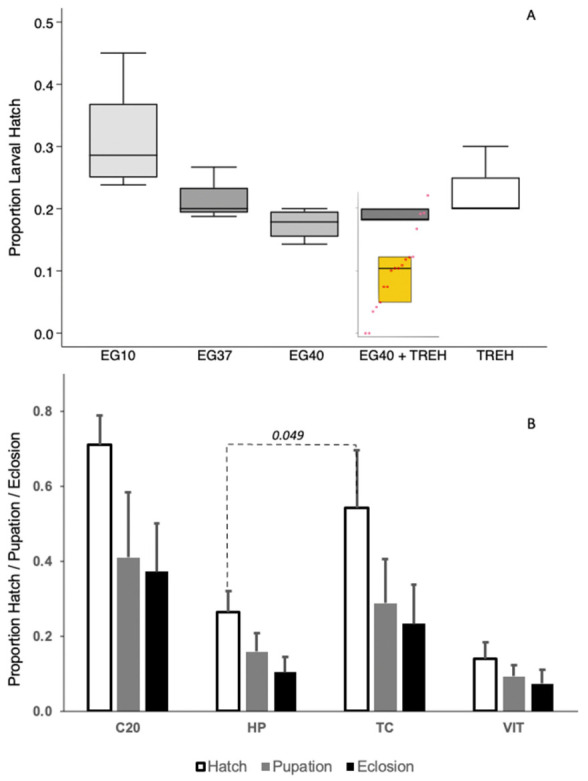
(**A**) Proportion of embryos that were viable after cryoprotectant treatment, as determined by the proportion of treated embryos that hatched, including cryoprotectant pretreatment with EG10—10% ethane diol (1.6 M), and followed by several vitrification solution treatments, namely EG37—37% ethane diol (5.96 M), EG40—40% ethane diol (6.45 M) or EG40+TREH—40% ethane diol + 0.5 M trehalose in Grace’s cell culture medium. Note that EG37, EG40 and EG40+TREH were all subjected to EG10 pretreatment. Post-vitrification viability of the embryos after retrieval from cryopreservation is represented by the inset yellow box graph and a strip chart. The embryos were loaded with 6.5 M of ethane diol and 0.5 M of trehalose prior to vitrification. (**B**) Proportion of embryos that hatched, larvae that pupated and moths that emerged from the embryos withdrawn during two separate sequential treatments. Treatment 1 was sodium hypochlorite (C20) and then heptane (HP). Treatment 2 was polysorbate 80 + sodium hypochlorite (TC) followed by vitrification (VIT).

Treatment with cryoprotectants reduced embryo survival in the following order. The initial incubation (often called cryoprotectant preloading) in 10% ethane diol (EG10; 1.6 M) in Grace’s medium exhibiting the highest survival, while the exposure to vitrification solution following the preloading step exhibiting increased mortality including the treatments of 37% ethane diol (EG37; 6 M), 40% (EG40; 6.45 M), 40% ethane diol with 0.5 M trehalose dihydrate, and 0.5 M of trehalose by itself (Figure 4A). The data are not adjusted for embryonic viability resulting from any of the previous steps, including exposure to 1.6 M ED. For the different vitrification mediums was not significantly different, H = 3.12, *p* = 0.078 & H = 4.5, *p* = 0.033) (Figure 4A). Interestingly, while permeability by either treating for 180 s in P80 containing sodium hypochlorite or 40 s of alkane treatment were not statistically different (Figure 3), these two treatments resulted in significantly different survival after retrieval from cryopreservation, with the mean proportion of permeabilized embryos that hatched after alkane treatment being ~50% less than the embryos from P80+hypochlorite treatment (HP hatch vs. TC hatch—H = 3.86, *p* = 0.049) (Figure 4B).

## 4. Discussion

This study adds to a growing body of evidence that demonstrates that the lack of a standardized cryopreservation procedure for lepidopteran embryos is due several factors, including developmental timing and the lack of synchrony in developing embryos, and more importantly, the incalcitrant nature of embryos to the various cryopreservation pretreatments, especially the dechorionation procedure. During the dechorionation procedure, the chorion is either destabilized or completely eroded away, primarily using chemical treatments. These embryos are now prone to dehydration and are amenable to xenobiotics, including the cryoprotective agents. However, this is not always the case [11,21,22,23,24].

As demonstrated for multiple dipteran and now lepidopteran species, the asynchronous development of embryos substantially impacts cryopreservation protocol development. Lower incubation temperatures have been noted to result in more synchronous development and eclosion [23]. Assessments of embryonic development at 21 °C and 24 °C were carried out with eyespot development and eclosion as developmental timepoint indicators. The embryos at 21 °C reached the eyespot stage approximately when the embryos at 24 °C began eclosing. The careful assessment of the morphology of the developing embryos indicated that cuticular development occurred prior to eyespot development and after completion of segmentation as well as the development of primary gut structures. Distinct segments were noted in the embryos after 30 h at 24 °C and ~50 h when incubated at 21 °C. Pigmented eyespots were observed at about 5–10 h after segmentations were noted. At 21 °C, the larval emergence occurred after 75–80 h, while the emergence was noticed after 55 h at 24 °C. However, the main aim of this exercise was to better synchronize the developmental stages and concentrate a specific developmental stage in higher numbers at specific timepoints. As noted above, lower incubation temperatures which are above the deleterious range for the species have been noted to result in more synchronous development and eclosion [23]. The synchronization phenomenon was not obvious in sunflower moth embryos incubated at the lower temperature of 21 °C compared with the ones incubated at 24 °C. The emergence times (slope of the sigmoidal curve in Figure 1) were approximately 50 h in both the incubation regimens. This could be due to the temperate climatic adaptation of sunflower moths versus the tropical adaptations among the tephritid flies. Despite the lack of increased synchronicity in embryonic development at lower temperatures, this assessment serves as a useful tool to approximate the time frame to process the embryos for cryopreservation. This data obtained in this study indicates that processing the 62–66 h old batch of embryos incubated at 21 °C or 34–35 h old embryos incubated at 24 °C would result in maximal embryonic survival after cryopreservation. As the temperature effect on embryonic developmental synchronicity was not noted, the generally used adult rearing temperature of 27 °C could also be used, and these embryonic batches would have to be permeabilized and cryoprotectant loaded approximately at 24–28 h prior to the embryonic development traversing the pigmented stage at ~30 h.

Rajamohan et al. [8] noted that the multilayered and crystalline chorion noted in most lepidopterans is the primary barrier that preserves embryos not only from desiccation and predation, but also from attempts to dechorionate the embryos using chemicals. Roversi et al. [7] reported that the adding of a detergent component to the dechorionation cocktail resulted in improved shrinkage and water uptake of embryos when challenged with 1 M sucrose [24]. Previously, when t-octylphenoxypolyethoxyethanol was assessed in *Pectinophora gossypiella* embryos, it was noted to be highly toxic at the concentrations that was tested then [8]. This was especially true when it was used in combination with a chorion-destabilization step. Studies on the hive moth *Galleria mellonella* have shown that polysorbate 80 (P80) is seemingly much less toxic/damaging to the embryos [24]. Hence, in this study, P80 was used to solubilize the hydrophobic components of the *H. electellum* embryos. In Rajamohan et al. [8], the hydrophobic surface of the lepidopteran eggs over the intact chorion was confirmed using ultrastructural studies where the layer was noticeable as an electron dense layer. While the same study also proposed a reverse permeabilization technique in which an alkane treatment preceded the dechorionation treatment to counter the supra-chorionic hydrophobicity, it was noted that the surfactant *p*-tert-octophenoxy polyethoxyethyl alcohol as a stand-alone permeabilization agent could not be used to permeabilize the embryos, as was determined by embryonic shrinkage or a reduction in supercooling points. However, sequential treatment with the surfactant followed by sodium hypochlorite could lead to permeabilization, albeit with a significant reduction in viability, possibly due to the higher concentrations of surfactants and sodium hypochlorite that were used. The current study used a mere 0.5% surfactant and 10% sodium hypochlorite but for an extended period of time of up to 180 s. Whether this mixture could be effectively used with *P. gossypiela* remains to be seen. In the case of *H. electellum*, however, this cocktail was sufficient to permeabilize the embryos enough for water and molecules of higher mass, such as 1,2-ethane diol (with a molecular weight of 62.04), to permeate the embryos, depending on their properties and configuration.

Another significant advancement in this study is the replacement of the arbitrary techniques for embryo permeabilization, such as shrinkage assessment and the probability of freezing point depression that were used in previous insect cryopreservation studies [25] with a more precise in vivo fluorescence intensity measurement of the permeated rhodamine B [26]. Additionally, while the freezing point depression method is a destructive method of estimation, the use of (semi-)vital staining allows for the further use of the embryo, including monitoring for embryonic development. Using the dye as an indicator is certainly a more nuanced approach. For instance, our previous study on *P. gossypiella* [8] relied on the embryonic shrinkage response to assess the effectiveness of permeabilization and noted that alkanes could indeed cause the embryo to be more amenable to water loss. However, this does not mean that the treated embryos could be loaded with sufficient levels of cryoprotectants. Nevertheless, in the present study, in term of the permeabilization, alkanes were very effective; however, they were not tolerated by the embryos, as noted by the lower hatch proportions of the treated embryos unlike previously noted in the case of dipteran embryos [23]. While Figure 3 shows that the embryonic dye uptake is higher with hexanes than heptane, Figure 2 reveals that the embryonic mortality is lesser after treatment with heptane.

The dye used in this study is capable of permeating lipid membranes and is useful for assessing the permeation, as well as the exudation, of water molecules through the biological membrane, and especially from multicompartmental systems such as the insect embryos [17,25]. The dye is, however, more mobile through lipids in the embryo, including the yolk, where water molecules are generally trapped in and out of the compartments [27]. This brings up a different issue of the presence of yolk and its effects on the CPA as well as water mobility in the late-stage embryos. In the case of dipterans, the egg yolk content is monitored, and as it is converted by the embryo to embryonic tissue the egg is loaded with CPA for cryopreservation. In Rajamohan et al. [23], yolk content reduction in the embryos of *Anastrepha ludens* to be as much as 69%. In the case of *Homoeosoma*, when the embryo was being CPA loaded, the yolk content was 73% and the embryonic proportion in the egg was 26%. Yolk is often considered an insurmountable barrier to CPA loading cryopreservation in general, as has been demonstrated in the past in insect, piscine and porcine systems [23,27,28].

The above stated critical issue of yolk content is also considered to be a major impediment to developing an embryonic cryopreservation procedure for the lepidopterans. Despite this, in past lepidopteran studies, a small but significant number of lepidopteran embryos from species such as *Spodoptera exigua*, *Galleria mellonella* and *Pectinophora gossypiela* have been shown to survive vitrification [6,7,8]. The careful assessment of the differences in the published protocols could be insightful to developing efficient but simpler procedures to cryopreserve the embryos of other lepidopteran species. The number of embryos that survived cryopreservation in the current study with *Homoeosoma* is similar to the numbers noted with *Pectinophora* vitrification studies, despite the modified permeabilization procedure. This observation is significant because the permeabilization protocol in the case of *Pectinophora* resulted in a higher hatch percentage (79.5 ± 14.3; [8]) than that noted in the current study, which used the modified procedure (70.1 ± 26.4%) despite the high variability inherent to the permeabilization treatment of insect embryos. Preliminary assessments using the previously reported reverse permeabilization technique resulted in <20% viable embryos (results not presented). This is probably due to the variation in chorionic morphology, as well as the chorionic and wax layer composition. *Homoeosoma* life stages, in general, have reasonably higher cold tolerances [5] than *Pectinophora,* and this could account for physio-morphological differences in the species that would also affect the cold tolerance characteristics and possibly the lipid composition in supra-chorionic as well as sub-chorionic layers [29].

The results not presented in this study also show that the supercooling points of *Homoeosoma* embryos were not significantly affected by any of the treatments, including the cryoprotectant loading. However, the water content was reduced by 23%. Our unpublished calorimetric studies on the dipterans, however, show a reduction of >45% of the water in CPA-loaded embryos (see, Supplementary Data S1). This brings forth the issue of the amount of CPA loaded into the embryo and the amount of water replaced from the embryo. Many recent studies on embryos in other species such as *Anopheles* mosquitoes indicate that permeabilized embryos loaded with cryoprotectants could be vitrified even with insignificant dehydration-driven shrinkage [30]. The conventional understating is that it is essential that the CPA permeates and replaces the free water in the embryos. However, this is not the case made by studies on dipteran embryos [23,31], where an equilibrium between the permeated cryoprotectant(s) and the dehydration afforded by non-permeating cryoprotectant(s) is seemingly sufficient to protect the embryos. While in the case of embryos that are in very early developmental stages, the permeation of the CPA to impact all the cells contained within the embryo is feasible; as noted with the above study with *Anopheles* embryos, current protocols for dipterans and lepidopterans use late developmental stages which are in the process of early organogenesis. It is nearly impossible to permeate all the cells with levels of cryoprotective agents sufficient to vitrify the permeated and dehydrated cytoplasm and the hemolymph in the various compartments. With dipterans, it has been shown that with careful observations of the embryonic developmental status and the precisely timed application of dehydration and cryoprotectant loading, one could vitrify the embryos even at freezing rates of ~5000 °C min^−1^ [15,23]. Therefore, with minimal dehydration and possibly lesser permeation of the CPA in the dipteran embryos than in the lepidopteran embryos, the rate of freezing might be more important. Late-stage embryos contain isolated compartments such as the gut, from where the osmotic derivation of water is substantially delayed. Loading the CPA into such less accessible sites will require prolonged equilibration and therefore will have higher deleterious effects such as CPA toxicity and osmotic shock in accessible sites. However, this could be compensated for by serial or multistep CPA loading, or by loading at lower temperatures and higher freeze and thaw rates by incorporating cryogens that are at temperatures below −196 °C, such as the slush nitrogen [17]. These are some of the aspects that are currently being assessed to improve the cryopreservation outcome for lepidopterans that lay smaller volumes of eggs.

## 5. Conclusions

In conclusion, this study illustrates the feasibility of vitrifying a lepidopteran embryo and attempts to comparatively illustrate the differences between this species and the previously reported species. While lepidopteran embryo cryopreservation studies are in the very early stages of advancement, the procedure used in this study to permeabilize the embryos significantly improved the proportion of embryos that hatched after cryopreservation to a maximum of 23%. Furthermore, the permeabilization step reported here is a single-step procedure and does not involve de-wetting or drying requirements. With nearly stable hatch proportions that could be obtained from two species of lepidopterans after cryopreservation, the possibility of adopting the protocol for additional lepidopteran species seems more likely. Considering the economical as well as the ecological role of various lepidopteran species, the need for germplasm conservation studies seems to be urgent.

## Data Availability

The data presented in this study are openly available in Kaggle at https://doi.org/10.34740/KAGGLE/DSV/4347004 [32].

## Supplementary Materials

The following supporting information can be downloaded at: https://www.mdpi.com/article/10.3390/insects13100959/s1, Supplementary Data S1, Table S1: Raw data for water content, Table S2: Means and Standard Deviations for water content and the embryonic supercooling points.
