# Supplementary material for: Vitrification of Lepidopteran Embryos—A Simple Protocol to Cryopreserve the Embryos of the Sunflower Moth, Homoeosoma electellum
Source: Insects. 2022 Oct 20;13(10):959. doi: 10.3390/insects13100959 (PMC9603988; doi:10.3390/insects13100959)
Supplement: Supplementary file 1 [file insects-13-00959-s001.zip › insects-1958566-supplementary.pdf]

Table S1: Raw data for water content

	Time(seconds)	Water Content per 100 embryos in microliters								
**NT**	NA	2.3	2.1	1.9	2.2	2.3	2.2	2.3	2.1	2.2
**TE**	180	2.3	2.2	2.2	2.2	2.4	2.1	2.5		
**TC**	180	2.7	2.3	2	2.4	2.4	2.6	2.9	2.5	2.7
**EG10**	600	2.1	1.8	1.9	2.4	2.1	2.1	1.9	1.9	
**EG37**	720	1.7	1.9	2.3	1.6	1.7	1.8			
**EG40+T**	720	1.2	1.4	1	1	1.5	1.2	1.4		

Table S2: Means and Standard Deviations for water content and the embryonic supercooling points

	Mean Water Content (µl)	Standard Deviation	Replicate count (n)	Mean ± SD Supercooling point (°C)
**NT**	2.2	0.13	9	-17.6 ± 4.8
**P80**	2.3	0.14	7	-16.3 ± 3.3
**P80+Clx**	2.5	0.26	9	-18.1 ± 6.1
**EG10**	2.03	0.19	8	-18.7 ± 7.0
**EG37**	1.8	0.25	6	-16.9 ± 3.4
**EG40+T**	1.24	0.19	7	-19.5 ± 2.8

Abbreviations

NT – Not treated; P80 – 0.5% Polysorbate 80; P80+Clx – P80 + 10% sodium hypochlorite; EG10 – 1,2-ethanediol (10%); EG37 – 1,2-ethanediol (37%); EG40+T – 40% 1,2-ethanediol in 0.5 molar trehalose; SD – standard deviation of the mean. ***Note***: all ethanediol and trehalose based solutions were mixed in Grace's insect cell culture medium.

Methods in brief: Tables S1 and S2 depict the same data set in raw and condensed form. The data was obtained by scanning the sunflower moth embryos in a differential scanning microcalorimeter at 1.5°C/min from 4°C to -40°C in a Perkin Elmer DSC7. The water content was derived from the area of integrated freeze exotherms of the embryos. Treated and untreated *Homoeosoma electelum* embryos that were airdried for 30 seconds were placed in a 50 microliter aluminum pan, covered in halocarbon oil (HC-700, Halocarbon corp., SC, USA) and sealed prior to being scanned.
